# A high-efficiency *Klebsiella variicola* H12-CMC-FeS@biochar for chromium removal from aqueous solution

**DOI:** 10.1038/s41598-021-85975-z

**Published:** 2021-03-23

**Authors:** Runlan Yu, Meilian Man, Zhaojing Yu, Xueling Wu, Li Shen, Yuandong Liu, Jiaokun Li, Mingchen Xia, Weimin Zeng

**Affiliations:** 1 School of Minerals Processing and Bioengineering, Central South University, Changsha, 410083 China; 2 Key Laboratory of Biometallurgy, Ministry of Education, Changsha, 410083 China

**Keywords:** Biochemistry, Environmental sciences

## Abstract

In polluted groundwater, surface water, and industrial sites, chromium is found as one of the most common heavy metals, and one of the 20 main pollutants in China, which poses a great threat to the ecological environment and human health. Combining biological and chemical materials to treat groundwater contaminated by heavy metals is a promising restoration technology. In this research, *Klebsiella variicola* H12 (abbreviated as *K. variicola*) was found to have Cr(VI) reduction ability. A high-efficiency *Klebsiella variicola* H12-carboxymethyl cellulose (abbreviated as CMC)-FeS@biochar system was established for Cr(VI) removal from aqueous solution. The Scanning Electron Microscope-Energy Dispersive Spectrometer (SEM–EDS), X-ray photoelectron spectroscopy (XPS) results indicated that CMC-FeS was successfully loaded onto the surface of biochar, and *K. variicola* H12 grew well in the presence of CMC-FeS@biochar with microbial biomass up to 4.8 × 10^8^ cells mL^−1^. Cr(VI) removal rate of CMC-FeS@biochar system, *K. variicola* H12 system and *K. variicola* H12 + CMC-FeS@biochar system were 61.8%, 82.2% and 96.6% respectively. This study demonstrated *K. variicola* H12-CMC-FeS@biochar system have potential value for efficient removal of Cr(VI) from Cr(VI)-polluted groundwater.

## Introduction

Groundwater as a significant part of water resources is very critical in the whole water circulation and even ecosystems. With the development of mineral resources, processing, and utilization, groundwater pollution has become increasingly serious such as acid mine drainage contained a lot of heavy metals^1^. It was reported that organic matter, heavy metals, and nitrates are the main pollutants in groundwater^2^. Some heavy metals cannot be degraded and stay in the environment for a long time^3,4^. In polluted groundwater, surface water, and industrial sites, chromium is found as one of the most common heavy metals, and one of the 20 main pollutants in China^5^. Cr(III) and Cr(VI) are the forms of chromium under normal circumstances. Under some special conditions, oxidation–reduction reactions may occur between trivalent chromium and hexavalent chromium. Trivalent chromium is considered to be the most stable form in the environment due to its low reactivity and biological toxicity^6^. However, the toxicity of hexavalent chromium is 100–1000 times that of trivalent chromium^7^. In industrial production, the discharged wastewater containing hexavalent chromium has the characteristics of carcinogen, mutagenicity, and bioaccumulation, which poses a great threat to the ecological environment and human health.

Physical and chemical methods such as electrocoagulation, ion exchange, reverse osmosis^8^ and biological methods are currently the main methods used in hexavalent chromium remediation. Some hydrogels-metal organic frameworks hybrid materials^9^, Microwave (MW)-assisted nanocomposite of magnetic graphene oxide functionalized tryptophan (MGO-Trp)^10^ and nanocomposite which based on crosslinked sodium alginate with iron oxide waste material^11^ are used to treat chromium. There is a very critical step in the treatment of pollutants containing chromate, which is the reduction process from hexavalent chromium to trivalent chromium^7,12^. Under the action of various reducing agents such as zero-valent iron (ZVI), sulfide, and humic acid, Cr(VI) can be reduced to Cr(III)^13,14^. Among them, iron sulfide nanoparticles are considered promising in the treatment of chromium-containing groundwater. Fe(II) and S(− II) have good reducing properties^15^. Compared with other sulfides, FeS has higher solubility and is easy to form Fe(OH)_3_–Cr(OH)_3_ precipitates. It has also demonstrated strong reducibility and other strengths^16,17^. Therefore, majority of researchers have paid more attention to the application of iron sulfide nanoparticles to the removal of Cr(VI)^18,19^.

As a reducing agent, iron sulfide can provide Fe (II) and S (− II) species that can effectively promote the reduction of hexavalent chromium. In addition, compared with other chemical reducing agents, it also has the advantages of high efficiency, low cost, and environmental friendliness. In recent years, biochar has been widely used as a stable solid carrier for dispersing nanoparticles due to its carbon-rich, porous and large surface area, promoting its application in environmental remediation^20–22^. The large specific surface area and abundant oxygen-containing functional groups in biochar are the main reasons for the improvement of its degradation efficiency in environmental remediation^23^. Biochar can be produced from solid wastes, such as agricultural residues, animal waste, and sludge, and is much cheaper than activated carbon. In addition, biochar can reduce Cr(VI) to Cr(III) by REDOX reaction of the surface functional group^24^. According to the report of Lyu et al.^25^, nano-FeS with biochar as a carrier has good effect in environmental applications and meets development needs.

Among the existing environmental remediation methods, physical and chemical methods are fast and effective. But the operation process is complicated. The equipment investment is expensive. And it will cause secondary pollution to the environment compared to biological remediation^26^. In recent years, bioremediation has become a new detox method for hexavalent chromium. It is considered as an alternative to physical and chemical methods and has significant benefits in environmental and ecological aspects. The effectiveness of bioremediation largely depends on the types of biological materials and microorganisms selected and their use^23^. Among them, microorganisms are considered to have potential in hexavalent chromium bioremediation due to their diversity, versatility, and environmental adaptability^27^. Microorganisms play an important role in chromium(VI) bioremediation. Thus, many microorganisms with good chromium(VI) removal ability have been isolated in recent decades, including *Bacillus subtilis*^28^, *pseudomonas putida*^29^, and the *genus* sp*.*^30^, *Rhodobacter* sp.^31^, *Desulfovibrio* sp.^32^, *Shewanella* sp.^33^. In addition, microorganisms also have the advantages of low cost and no secondary pollution to the environment. Therefore, the bioremediation technology of chromium by bacteria has attracted the attention of many researchers^34,35^.

In previous researches, there are more studies on surface water, while groundwater has received less attention than surface water. The oxygen content in groundwater is relatively small. The *K. variicola* H12 are facultative anaerobic bacteria. However, in the reported works, there are few studies on the effectiveness of *K. variicola* H12 in the removal of Cr(VI). At the same time, carboxymethyl cellulose (abbreviated as CMC)-FeS@biochar was effective and cheap in Cr(VI)-contaminated sites. So, the *K. variicola* H12-CMC-FeS@biochar system was constructed to remove Cr(VI) for the first time in this study, and evaluated the influence of environmental factors on the Cr(VI) removal of *K. variicola* H12-CMC-FeS@biochar system.

## Materials and methods

### Chemicals

All chemicals used in this work were of analytical grade. Sodium sulfide nonahydrate (Na_2_S•9H_2_O) was purchased from Wenjiang Chemical Technology. Iron sulfate heptahydrate (FeSO_4_·7H_2_O), 1, 5-diphenyl carbazide, FeS, carboxymethyl cellulose (abbreviated as CMC), Phosphoric acid, potassium permanganate(KMnO_4),_ urea (NH_2_)_2_CO, sodium nitrite(NaNO_2)_, NaCl, K_2_Cr_2_O_7_, C_6_H_12_O_6_, C_12_H_22_O_11_, (C_6_H_10_O_5_)n, C_2_H_3_O_2_Na·3H_2_O, CH_4_N_2_O, NaH_2_PO_4,_ and Na_3_PO_4_ were provided from Sinopharm Chemical Reagent Co., Ltd (Shanghai, China). Sulfuric acid were supplied by Xilong Science Chemical Technology. The Beef Extract, Peptone, Tryptone and yeast extract were purchased from shanghai Macklin Biochemical Co., Ltd (Shanghai, China). (NH_4_)_2_SO_4_ and NH_4_H_2_PO_4_ were supplied by Damao Chemical Reagent Co., Ltd (Tianjin, China). KH_2_PO_4_ was purchased from Hengxing Chemical Reagent Co., Ltd (Tianjin, China). Dissolve 2.0 g K_2_Cr_2_O_7_ in 1 L of deionized water to obtain a Cr(VI) mother solution with a final concentration of 2000 mg L^−1^.

### *K. variicol*a H12

*Klebsilla variicola* H12 was obtained from our laboratory, which was screened from sludge in a previous work^1^, Based on the blasted results of the 16S rDNA gene sequence, this novel strain was named as *Klebsiella variicola* H12. It was preliminarily identified by using physiological and biochemical analysis including colony morphology, Gram staining, methyl red (MR), oxidase, Voges Proskauer test (VP), nitrate reduction, malonate utilization. The experiment was based on LB medium(10 g L ^−1^ Tryptone, 5 g L ^−1^ yeast extract, 10 g L ^−1^ NaCl), with different carbon sources (C_6_H_12_O_6_, C_12_H_22_O_11_, (C_6_H_10_O_5_)_n_, C_2_H_3_O_2_Na·3H_2_O), different nitrogen sources (Beef Extract, Peptone, (NH_4_)_2_SO_4_, CH_4_N_2_O) and different organic salts(NaH_2_PO_4_, KH_2_PO_4_, NH_4_H_2_PO_4_, Na_3_PO_4_, Nacl) as medium components. The energy substrate experiment was performed with 150 rpm at 30 °C for 24 h. In addition, the experiments set gradient pH (2.0, 4.0, 6.0, 8.0, 10.0) and temperature (20 °C, 30 °C, 40 °C, 50 °C, 60 °C) in LB medium at 150 rpm. The incubation time was the same as above. The above experiments were set up in two parallel groups.

### Removal of Cr(VI) by *K. variicola* H12

*K. variicola* H12 was cultured with 150 rpm at 30 °C in LB medium for 20 h to obtain late logarithmic phase cells. 10 g L^−1^ Tryptone, 5 g L^−1^ yeast extract, 10 g L^−1^ NaCl were prepared into a liquid medium, and the pH value was adjusted to 6.0. In the experimental group, the mother solution of K_2_Cr_2_O_7_ with a concentration of 2000 mg L^−1^ was added to the LB medium, so that the final concentration of Cr (VI) in the medium reached 20 mg L^−1^, 60 mg L^−1^, 100 mg L^−1^, 200 mg L^−1^. Control group was cultured in the medium without the mother solution of K_2_Cr_2_O_7._ 1 mL samples were taken out every two hours, and the cell density of *K. variicola* H12 was measured with an Epoch Microplate spectrophotometer (BioTek Instruments, Inc), at OD_600_ (optical density at 600 nm)^36^. Then, the concentration of Cr(VI) in sample was determined by 1, 5-diphenyl carbazide method^37^. Total chromium was first oxidized by potassium permanganate and then measured. Specifically, 0.2 g of 1,5-diphenylcarbazide was dissolved in 50 mL acetone, then 50 mL of deionized water, 12.5 mL of 85% phosphoric acid and 12.5 mL of 95% concentrated sulfuric acid were mixed with the solution to obtain a chromogenic reagent. Determination of total chromium is performed by adding 40 g L^−1^ KMnO_4,_ 200 g L^−1^ (NH_2_)_2_CO and 20 g L^−1^ NaNO_2_ before that step. 3.0 mL of the chromogenic reagent was added to the as-mentioned supernatant and the mixed solution was tested at 540 nm on a spectrophotometer.

### Preparation of CMC-FeS@biochar

CMC-FeS@biochar was prepared following the methods used by Lyu et al.^25^. Biochar originated from wheat straw powders and nanometer materials CMC-FeS were used to form CMC-FeS@biochar. In short, the wheat straw powder is pyrolyzed in a muffle furnace [Hefei Kejing Materials Co., Ltd. (Anhui, China)] at 600 °C for 2 h (under oxygen-limited conditions) to obtain biochar for subsequent use in the production of CMC-FeS@biochar. The preparation process of CMC-FeS@biochar was as follows: In the N_2_ purging solution, 0.870 g of FeSO_4_·7H_2_O is dissolved in 500 mL deionized water and deoxygenated with N 2 (> 99%) for 1 h. Then 27.5 mL of 1% CMC solution was added to the above solution. Next, 275 mg biochar is introduced into the mixture under strong magnetic stirring for 1 h. Finally, 22.5 mL Na_2_S solution (0.751 g Na_2_S·9H_2_O) was added to the complex.

### Effect of CMC-FeS@biochar on the growth of *K. variicola* H12

Before we used the mixture of H12 and CMC-FeS@biochar to remove Cr(VI), the effect of the chemical adsorbent (CMC-FeS@biochar) on the growth and tolerance of *K. variicola* H12 was tested. The growth experiments were carried out in 250 mL Erlenmeyer flasks. The flasks containing 100 mL of LB medium and 2 mL K*. variicola* H12 (logarithmic phase) were amended with CMC-FeS@biochar at 0.25 g L^−1^. The Erlenmeyer flasks were then incubated at 30 °C and 150 rpm on a thermostatic culture oscillator for 24 h (ZWY-2102C, Zhicheng analytical Instrument Co., Ltd., Shanghai, China). Control tests were conducted in the absence of adsorbents/chemical reductants under identical conditions. In addition, four different chromium concentrations (20 mg L^−1^, 60 mg L^−1^,100 mg L^−1^,200 mg L^−1^) were set up for tolerance experiment. The culture conditions were the same as the above growth experiments. The water phase and the solid were separated every 2 h and settled by gravity for 15 min^25^. After that, to determine the cell density and the OD_600_ of *K. variicola* H12, 300 μL of the sample was taken out of the Erlenmeyer flask. At 24 h, the control group was collected after centrifugation at 5000 rpm for SEM observation.

### Removal of Cr(VI) by *K. variicola* H12-CMC-FeS@biochar system

In this batch of experiments, *K. variicola* H12 in the late logarithmic phase cells were selected. Seven groups of single bacteria group, chemical material group (biochar, FeS, CMC-FeS@biochar), mixed of *K. variicola* H12 and chemical materials group (*K. variicola* H12 + biochar, *K. variicola* H12 + FeS, *K. variicola* H12 + CMC-FeS@biochar) were set up. All batch experiments used 250 mL Erlenmeyer flask incubated in Incubation Oscillator. The Erlenmeyer flask contained 20 mg L^−1^ of sterile Cr(VI) solution. In addition, a series of experiments was carried out to determine the effects of oxygen (aerobic/anaerobic), pH (4.0–8.0) and inoculation amount (2–10%) on Cr(VI) removal in collaborative system. Initial solution pH (6.0) was not modified. In addition, SEM analysis were conducted on the stability of *K. variicola* H12-CMC-FeS@biochar under different acidic and alkaline conditions before exploring the pH value. In these experiments, except that chromium is not added to the medium, other conditions are consistented with the above experiments to explore the effect of pH on the treatment effect of *K. variicola* H12-CMC-FeS@biochar. The concentration of Cr(VI) in the sample was measured at regular intervals. Real water sample collected from the leachate around a leather factory in Changsha, China, which experimental condition was consistent with the above *K. variicola* H12-CMC-FeS@biochar system. Regeneration of exhausted *K. variicola* H12-CMC-FeS@biochar saturating with Cr(VI) was conducted by HCl (0.1 mol L^−1^)^38^. 40 mL of desorbent and recovered adsorbent are stored in a 250 mL Erlenmeyer flask, and shaken continuously for 100 min at 150 rpm and 30 °C, then determined the concentration of Cr(VI) in the filtrate. The specific measurement method of Cr(VI) has been shown in “Removal of Cr(VI) by *K. variicola* H12” of this article.

### Analytical methods

The late logarithmic phase cells were centrifuged at 8000 rpm for 5 min, and then washed three times with phosphate buffer. The morphologies of bacterial cells and CMC-FeS@biochar were observed by XL 30ESEM scanning electron microscopy (SEM) (Hitachi S4800)^39^. The energy dispersive spectroscopy (EDS) at a voltage of 150 keV was also operated to identify the chemical elements on cell and CMC-FeS@biochar surface. In addition, before adsorption and after adsorption of Cr(VI), the functional groups was analyzed by the Fourier transform infrared spectrophotometry (FTIR spectrum, Nexus670, Thermo Nicolet Co.) with the wavelength range of 400–4000 cm^−1^. X-ray photoelectron spectroscopy (XPS) of CMC-FeS@biochar was done before adsorption of Cr(VI). The XPS spectra operated on a Thermo Fisher-VGScientific (ESCA-LAB 250Xi) photoelectron spectrometer. The surface area and pore structures of CMC-FeS@biochar were determined by the BET adsorption–desorption method (version 11.02; Quantachrome Instruments, USA).

## Results and discussion

### *Klebsiella variicola* H12

#### Physicochemical property of *K. variicola* H12

**Figure 1:**
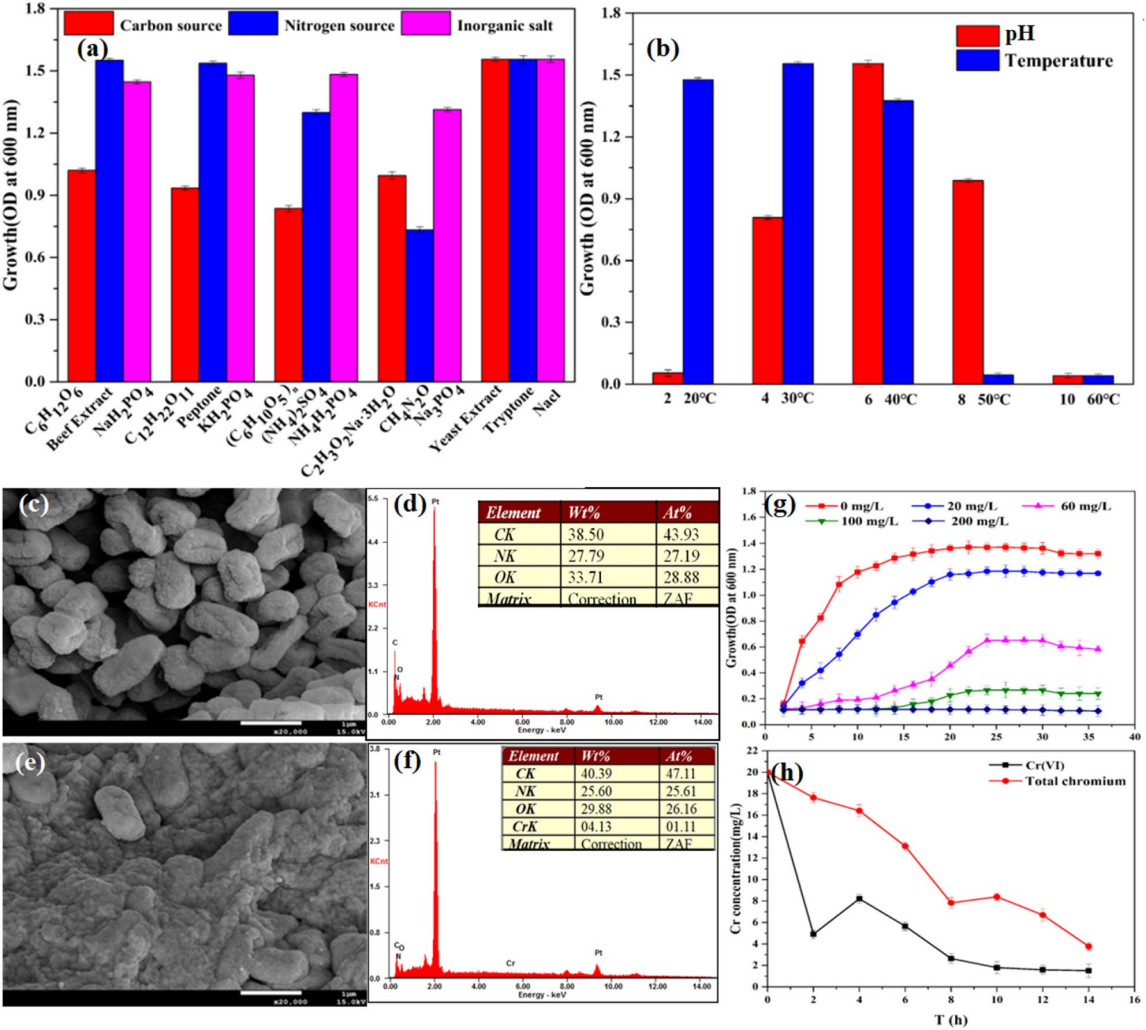
Optimization of growth conditions for *K. variicola* H12 (**a**) Energy substrate (**b**) pH and temperature; SEM–EDS analysis of (**c**, **d**) K. variicola H12 cultured with Cr(VI)-free and (**e**–**f**) K. variicola H12 cultured with Cr(VI) in LB medium for 24 h. (**g**) Effect of Cr(VI) concentrations on the growth of *K. variicola* H12 in LB medium. Experimental conditions: growth determined by cell turbidity measured at 600 nm. (**h**) Cr(VI) reduction experiment by *K. variicola* H12.

The Physicochemical property of *K. variicola* H12 and the utilization of energy substrates are shown in Fig. 1. When culture medium was pH 6.0 and the temperature was 30 °C, the growth of the strain was the best which OD_600_ reached around 1.6 (Fig. 1b) (Data provided in Table S2a,b). Therefore, subsequent experiments could choose this condition to cultivate the strain. At the same time, *K. variicola* H12 was negative by Gram stain. Physiological and biochemical of *K. variicola* H12 identification results shown that: the nitrate reduction test, VP test, malonate test, citrate test and urease test were positive. The oxidase test, indole test and methyl red test were negative (Table 1) (+ :Positive reaction; −:Negative reaction). The energy used by the bacteria is shown in Fig. 1a (Data provided in Table S1a–c). The results shown that the strain could use carbon sources such as C_6_H_12_O_6_, C_12_H_22_O_11_, (C_6_H_10_O_5_)n, C_2_H_3_O_2_Na·3H_2_O, nitrogen sources such as Beef Extract, Peptone, (NH_4_)_2_SO_4_, CH_4_N_2_O, and inorganic salts such as NaH_2_PO_4,_ KH_2_PO_4_, NH_4_H_2_PO_4_, Na_3_PO_4_, Nacl to meet the needs of spontaneous growth. However, in the culture medium that tryptone as nitrogen sources, yeast extract as the carbon source, and NaCl as the inorganic salt, the strains grew best and could be used as the culture medium for subsequent experiments.

**Table 1:** Physiological and biochemical characteristics of *K. variicola* H12.

Characteristics	Results
Methyl-red	**–**
Oxidase	**−**
VP	**+**
Nitrate reduction	**+**
Citrate utilization	**+**
Malonate utilization	**+**

#### SEM–EDS analysis of *K. variicola* H12

The analyses of SEM–EDS were adopted to investigate the cell morphological changes of *K. variicola* H12 before and after treated with 20 mg L^−1^ Cr(VI) for 24 h (Fig. 1c–f). It can be seen that in the Cr(VI) -free culture, the cells were short rod shaped [size (0.5 ~ 1) μm × (1 ~ 2) μm] and the surface was depressed (Fig. 1c). According to EDS analysis (Fig. 1d), C, N, O were the main elements of *K. variicola* H12. After adding Cr(VI) to the culture medium, the morphology of *K. variicola* H12 cells changed (Fig. 1e). Large number of cells were covered with a certain substance. EDS analysis (Fig. 1f) further proved that the main elements on the surface of these substances contained C, N, O and Cr is also detected. It was speculated to be some organics such as Extracellular polymers secreted of strain after being stimulated by chromium^40–43^. This was one of the stress response of strains to the surrounding environment.

#### Cr(VI) reduction by *K. variicola* H12

The effect of different concentrations of chromium on the growth of *K. variicola* H12 is shown in Fig. 1g (Data provided in Table S3). From the change rule of OD_600_, it can be seen that the value of OD_600_ gradually decreased with the increase of Cr(VI) concentration in the same culture time. It indicated that the biotoxicity of metals increased with the increase of metal concentrations. In the absence of Cr(VI) solution, the bacteria have the highest activity in the culture medium, and the OD_600_ reaches 1.4 after 20 h of culture. Before this period, cell synthesis and metabolism were very rapid due to sufficient nutrients in the LB medium and no toxic effects of heavy metals. When the Cr(VI) concentration increased from 20 to 100 mg L^−1^ (20 mg L^−1^, 60 mg L^−1^, and 100 mg L^−1^), the maximum OD value decreased from 1.2 to 0.2. When Cr(VI) concentration reaches 200 mg L^−1^, *K. variicola* H12 did not grow. It illustrated that the *K. variicola* H12 could survive in Cr(VI) concentration of < 200 mg L^−1^, which proved that the strain has the ability to tolerate Cr(VI). The toxic effect of heavy metals in the solution increased with the gradual increase of chromium concentration and the growth and reproduction ability of the strain decreased. Under high Cr(VI) concentration, the growth and reproduction of *K. variicola* H12 are inhibited. In addition, In order to verify that *K. variicola* H12 has ability to remove Cr(VI) from liquid culture medium, the concentration of Cr(VI) in 20 mg L^−1^ culture medium was analyzed every 2 h. As shown in Fig. 1h (Data provided in Table S4), the reduction rates of Cr(VI) were 58.96%, 86.79% and 93.87% at 4 h, 8 h and 12 h after bacterial cells treated. At any time point, the total chromium content that can be detected in the solution was higher than the Cr(VI) content. It indicated that Cr(III) was present in the solution, the concentration Cr(III) provided in Table S4. Cr(III) may be derived from the reduction of Cr(VI) by reductase which produced by bacteria in the growth and metabolism of Cr(VI) environment.

### Characterization of *K. variicola* H12-CMC-FeS @ biochar system

#### XPS and surface area analysis of CMC-FeS@biochar

**Figure 2:**
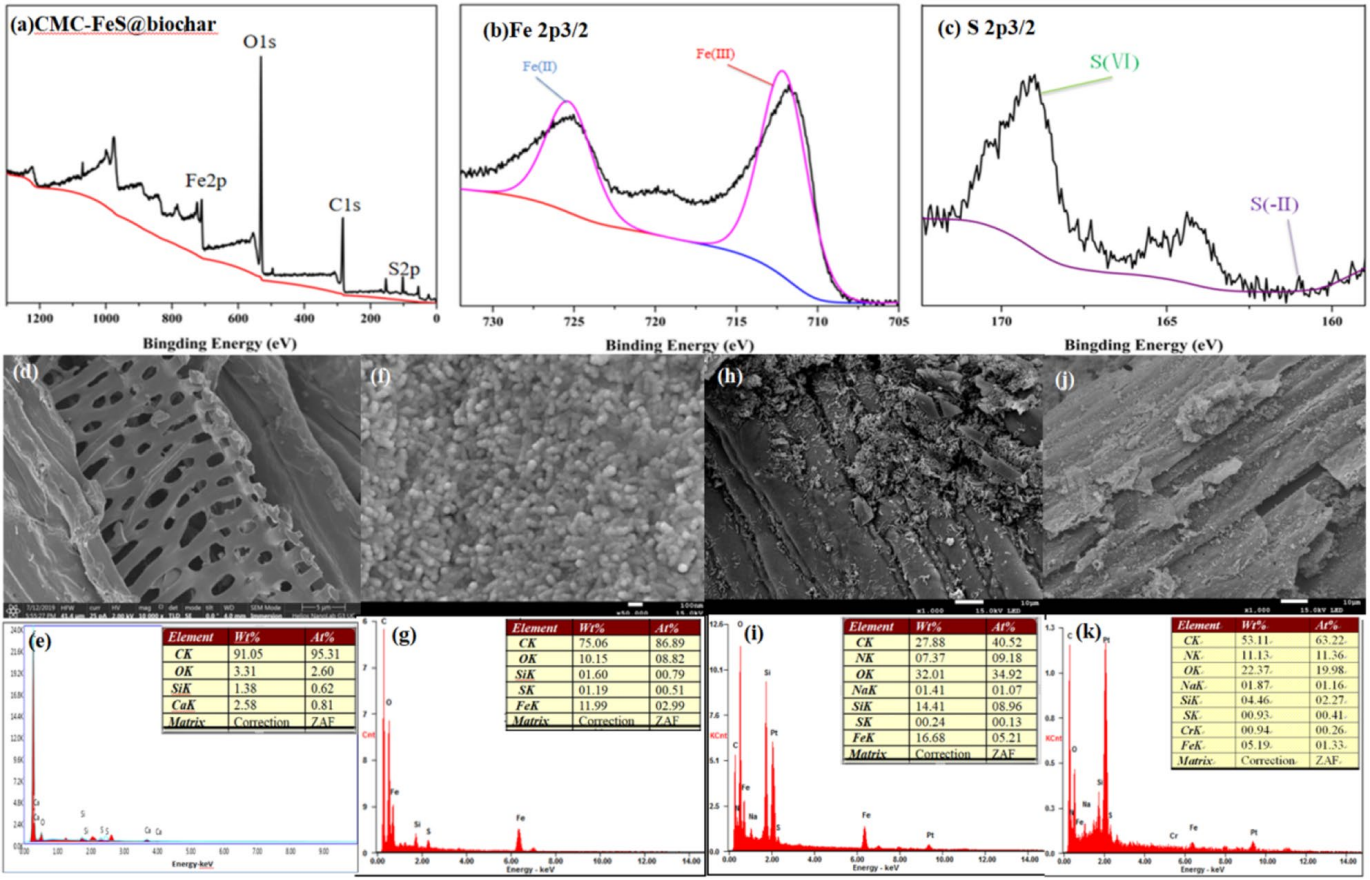
XPS analysis for (**a**) CMC-FeS@biochar, (**b**) highresolution Fe 2*p* XPS spectrum, (**c**) high resolution S 2*p* XPS spectrum. SEM–EDS analysis of (**d**–**e**) biochar, (**f**–**g**) CMC-FeS@biochar, (**h**–**i**) CMC-FeS@biochar and *K. variicola* H12. (**j**–**k**) CMC-FeS@biochar and *K. variicola* H12 after treating chromium.

XPS analysis for CMC-FeS@biochar and high resolution spectra of the Fe 2p and S 2p region after loading were presented in Fig. 2a–c. The main elements contained in CMC-FeS@biochar were carbon (47.18%), oxygen (43.95%), iron (4.67%) and sulfur (1.94%)(Fig. 2a). According to the reported literature, the binding energies of 711.1 eV and 725.1 eV represent Fe^3+^2*p*_3/2_ and Fe^2+^2*p*_1/2_^44,45^ (Fig. 2b) respectively, and the satellite between the two dominant peaks is contributed by Fe^2+^ through the process of oscillation^46,47^. The binding energies of 169.1 eV and 160.9 eV represent S^6+^2*p*_3/2_ and S^2–^2*p*_3/2_^45,48^ (Fig. 2c). It also indicated that FeS was successfully attached onto the surface of biochar. In addition, as shown from Table 2 (BET surface area calculated in the relative pressure region P/P0 = 0.300, Micropore volume determined at P/P0 = 0.991, Average pore diameter obtained from BJH equation using N_2_ isoterms). After adsorbing Cr(VI), micropore volume and average pore diameter decreased, which indicated the existence of pore filling effect during the process.

**Table 2:** Structure properties of *K. variicola* H12-CMC-FeS@biochar before and after treatment of Cr(VI).

Sample	SBET (m^2^ g^−1^)	Micropore volume (cm^3^ g^−1^)	Average pore diameter (nm)
*K. variicola* H12-CMC-FeS@biochar	61.108	0.083	2.955
*K. variicola* H12-CMC-FeS@biochar + Cr(VI)	16.157	0.056	2.586

#### SEM–EDS analysis of *K. variicola* H12-CMC-FeS @ biochar system

**Figure 3:**
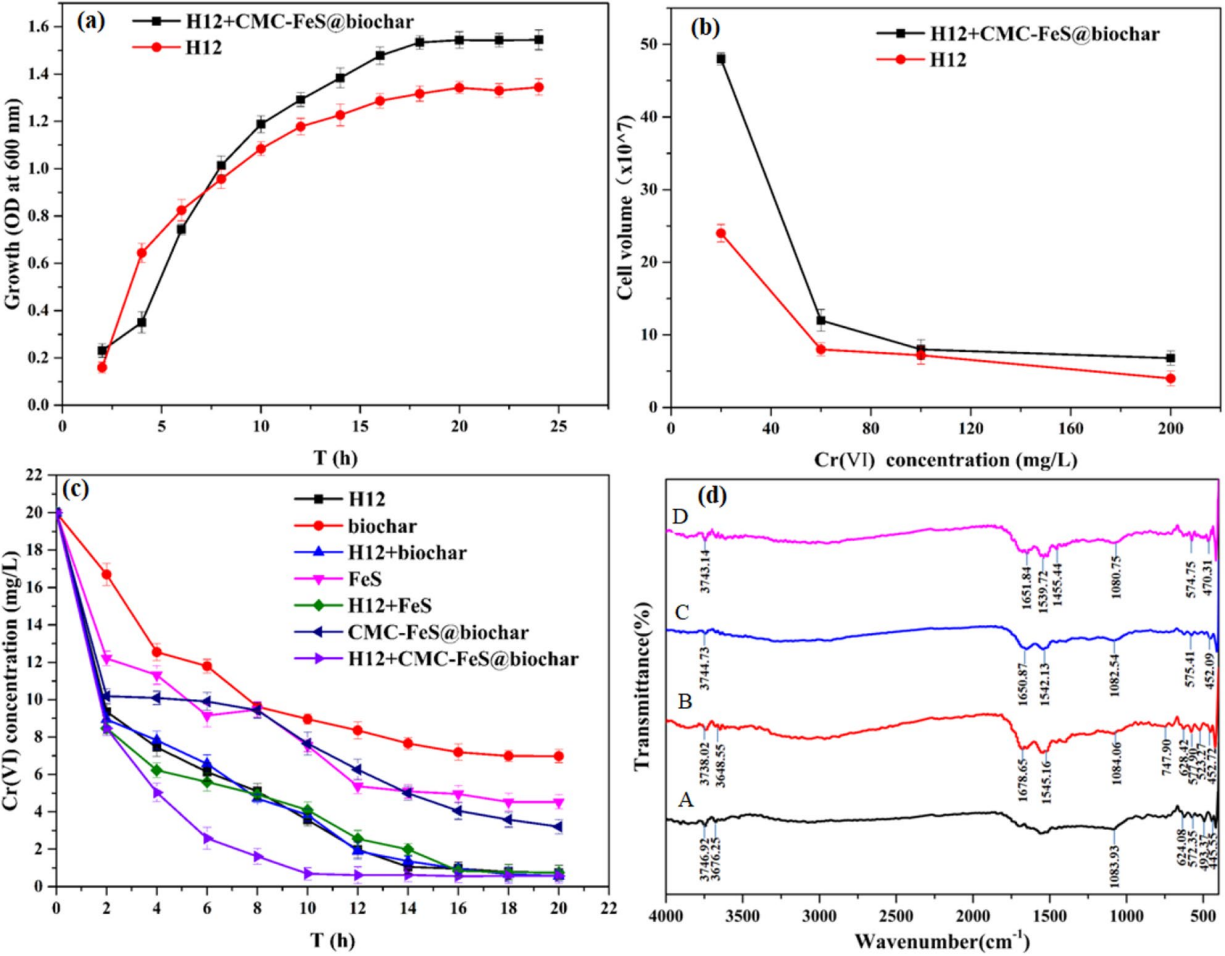
(**a**) Effect of CMC-FeS@biochar, (**b**) Cr(VI) concentration on the growth of *K. variicola* H12 in LB medium for 24 h. Experimental conditions: growth determined by cell turbidity measured at 600 nm. (**c**) Cr(VI) removal efficiency of H12, biochar, H12 + biochar, FeS, H12 + FeS, CMC-FeS@biochar, H12 + CMC-FeS@biochar. Experimental conditions: Cr(VI) concentration 20 mg L^−1^, solution volume 100 mL, and reaction time 24 h. (**d**) FTIR spectra of (A)CMC-FeS@biochar and compound of *K. variicola* H12 and CMC-FeS@biochar being treated with 20 mg L^−1^ Cr(VI) under different periods (B) 4th, (C) 10th, (D) 16th h, respectively. All the above experiments are carried out at pH 6.0.

SEM–EDS analysis of the bare biochar, CMC-FeS@biochar, *K. variicola* H12-CMC-FeS@biochar composite are shown in Fig. 2d–k. Morphological observations shown that the biochar (Fig. 2d.) was porous and rough, that resulted in more uniform attachment of FeS and creation of larger specific surface area and pore volume yielding more sorption sites. Compared with biochar, clearly defined aggregated particle was observed on the surface of biochar (Fig. 2f). It indicated that irregular granular like CMC-FeS@biochar particles in diameter of < 100 nm were attached to the biochar surface. The analysis of EDS was adopted to investigate the elemental in CMC-FeS@biochar composite. As seen from Fig. 2e, C, O, Si, Ca were detected by EDS which should be ascribed to the components of the biochar. After supporting, the element Fe and S were detected (Fig. 2g). The successful loading of FeS on the surface of biochar was initially proved. In *K. variicola* H12-CMC-FeS @ biochar system, SEM image of *K. variicola* H12-CMC-FeS@biochar shown in Fig. 3e,f. The *K. variicola* H12 was unevenly attached to the surface of the CMC-FeS@biochar (Fig. 2h). As seen from Fig. 2i, when a large amount of *K. variicola* H12 was adsorbed on the surface of the material, EDS analysis of the surface of the material detected the presence of N element. It indicated that CMC-FeS@biochar has formed a complex with *K. variicola* H12. As shown in Fig. 2k, chromium was found on the surface of CMC-FeS@biochar. It indicated that there is an adsorption effect between CMC-FeS@biochar and chromium.

### Removal of Cr(VI) by *K. variicola* H12-CMC-FeS@biochar system

#### Effect of CMC-FeS @ biochar on the growth and tolerance of *K. variicola* H12

The effect of CMC-FeS @ biochar on the growth and tolerance of *K. variicola* H12 are shown in Fig. 3. The growth of *K. variicola* H12 on LB medium was assessed in the presence of CMC-FeS@biochar (Fig. 3a) (Data provided in Table S5). The stabilization of CMC (CMC-FeS) and supporting of biochar (CMC-FeS@biochar) reduced the toxicity of FeS, as evidenced by increasing the growth of *K. variicola* H12 from OD 1.39 for FeS to 1.57 in the late log phase, respectively. The CMC-FeS@biochar increased the growth of *K. variicola* H12 at late logarithmic phase, though it decelerated the growth of *K. variicola* H12 at lag phase. These was consistent with the research results of Satyendra et al.^25^. Bare FeS has significant inhibitory effect on bacterial^25^ and the study of Lee et al. also reported that bacterial growth was restricted by iron nanoparticles through physical coating, oxidative stress, and membrane disruption^49^. The effect of CMC-FeS@biochar on the tolerance of *K. variicola* H12 is shown in Fig. 3b (Data provided in Table S6). At a lower Cr(VI) concentration (20 mg L^−1^), the effect of CMC-FeS@biochar on the growth of bacteria was significantly higher than that of high concentrations obviously. When CMC-FeS@biochar was present, the highest cell concentration of bacteria can reach to 4.8 × 10^8^ cells mL^−1^. It indicated that CMC-FeS@biochar promoted the growth of *K. variicola* H12 and lay the foundation for the efficient removal of Cr(VI) in groundwater.

#### Performance of *K. variicola* H12-CMC-FeS@biochar system in removing Cr(VI)

Cr(VI) can be removed by *K. variicola* H12-CMC-FeS@biochar system (Fig. 3c) (Data provided in Table S7a,b). The experiments are carried out at pH 6.0. In general, Cr(VI) removal rate followed the order: mixed of *K. variicola* H12 and chemical materials group (*K. variicola* H12 + biochar, *K. variicola* H12 + FeS, *K. variicola* H12 + CMC-FeS@biochar) > single bacteria group > chemical material group (biochar, FeS, CMC-FeS@biochar). Compared with chemical material group (biochar, FeS, CMC-FeS@biochar). When the experiment was conducted for 10 h, the Cr(VI) removal rate of CMC-FeS@biochar system, single bacteria system and bacteria + CMC-FeS@biochar system were 61.8%, 82.2% and 96.6% respectively. The study of Lyu et al. shown that CMC-FeS@biochar surface sorption and reduction were the dominant removal mechanisms during the chromium removal process^15^. While in reduction, the removal mechanism of Cr(VI) mainly relied on the reducibility of Fe^2+^ and S^2–15^. The dominant removal mechanisms of *K. variicola* H12 on Cr(VI) was confirmed in 3.1 (Fig. 1h). In addition, a small amount of surface adsorption has also been confirmed in 3.1 (Fig. 1e,f). In the bacteria + CMC-FeS@biochar system, the removal efficiency of Cr(VI) was improved, compared to the CMC-FeS@biochar and single bacteria treatment system. These showed that in the mixed system, CMC-FeS@biochar and *K. variicola* H12 have a synergistic relationship with each other when processing Cr(VI). But it is not a superposition of efficiency. Compared with chemical group [Cr(VI) removal efficiency were less than 62.3%] and the bacteria mixed with chemical group (the Cr(VI) removal efficiency of *K. variicola* H12 + biochar, *K. variicola* H12 + FeS reached 80.8%, 79.5% respectively), the bacteria + CMC-FeS@biochar system removal rates of Cr(VI) increased by 1.6 times and 1.2 times respectively. In the 6th hour, the removal rate of Cr(VI) reached the highest value first. When the same amount of heavy metal Cr(VI) was processed, the other experimental groups had a longer processing time and a relatively low removal rate. At the same time, Compared with the research by Liu et al.^50^ (Who used Cr (VI) resistant bacterial strains to treat chromium-containing sludge, the Cr (VI) level was decreased by 90% within 65 h, the processing time is shortened by about 1/6, and the efficiency is 1.05 times of them in this study). In situ remediation, nZVI and other chemical methods were used by some researchers and the efficiency can reach more than 95%^51^. However, the extensive use of these chemicals caused secondary pollution to the surrounding environment. The superiority of *K. variicola* H12-CMC-FeS@biochar system to treat Cr (VI)-contaminated groundwater was confirmed.

For further application of *K. variicola H12*-CMC-FeS@biochar in wastewater treatment, a regeneration study was performed in this work. There was a gradual decrease in Cr(VI) desorption rate with an increasing number of cycles as shown in Fig. S1. The desorption rate of cycles 1, 2, 3 and 4 were found to be 61.5%, 40.6% 26.7% and 11.2% respectively. It was observed that cycle 1 showed good retrieval. Cr(VI) removal decreased from 96.6% in LB medium to 50.4% in real water sample (Fig. S2). It is speculated that some inorganic and organic substances in the real water sample have an inhibitory effect on the removal of chromium^52^.

#### FTIR analysis of *K. variicola* H12-CMC-FeS@biochar system

FTIR can be used to understand the types of functional groups contained in the substance. Before and after biochar treatment of chromium, FTIR analysis (Fig. S3) found that a characteristic and broadband appeared around at 3200–3420 cm^−1^ (Stretching vibration of −OH)^53^, 1080–1153 cm^−^1 (bending vibrations of −COOH)^54^, around 796 cm^−1^ (vibration of Si–O–Si)^55^. Some new characteristic and broadband appeared around 1450–1460 cm^−1^ (deformation vibration of CH_2_), 1400–1410 cm^−1^ (Stretching vibration of C=O)^53^, around 2960 cm^−1^ (corresponding to symmetric stretching vibration C–H)^56^. By comparing the changes of functional groups at different time points, the reaction of *K. variicola* H12-CMC-FeS @Biochar compound was further analyzed. The FTIR spectra of CMC-FeS@biochar and *K. variicola* H12-CMC-FeS@biochar being with 20 mg L^−1^ Cr(VI) under different reaction time were presented in Fig. 3d. The major bands of CMC-FeS@biochar (spectra A) can be assigned as follows: A characteristic and broadband appeared around 3738–3744 cm^−1^ (the N–H stretching vibrations)^46^, 1080–1084 cm^−1^ (the symmetric stretching of C–O–C)^57,58^, the characteristic absorption peak appears in 574–577 cm^−1^ attributed the vibration of iron oxide Fe–O^57^. After adding strains to compound, some new characteristic and broadband appeared around 1650–1678 cm^−1^ (C=O groups from the −COOH)^57^, 1539–1545 cm^−1^ (the bending vibration of C–N)^42^. These indicated that the sites available for binding were increased with *K. variicola* H12. In the *K. variicola* H12-CMC-FeS@biochar compound (spectra B–D), as the reaction time changed, the functional group N–H, C=O, C–N, C–O–C, Fe–O were shifted. Among them, N–H, C=O were shifted greatly within 4–10 h (spectra B–C) and stabilized within 10–16 h (spectra C–D). C–N were shifted uniformly in 4–10 h, C–O–C, Fe–O displacement trend was the same. These showed that N–H, C=O, C–N, C–O–C, Fe–O were involved in the chromium adsorption and reduction process. In the *K. variicola* H12-CMC-FeS@biochar complex, the functional groups of both *K. variicola* H12 and CMC-FeS@biochar still appeared in the infrared spectrum of the complex. Based on the results of the previous “Performance of *K. variicola* H12-CMC-FeS@biochar system in removing Cr(VI)” (the treatment of chromium by the compound is better than that of *K. variicola* H12 and CMC-FeS@biochar alone), it can be further speculated that in the process of processing chromium, there may be a cooperative processing relationship between *K. variicola* H12 and CMC-FeS@biochar. Comparing the spectra of biochar and *K. variicola* H12-CMC-FeS@biochar, *K. variicola* H12-CMC-FeS@biochar provided more functional groups for binding Cr(VI) and has a better ability to loading Cr(VI).

### Effects of inoculation amount, oxygen condition and solution pH on Cr(VI) removal

**Figure 4:**
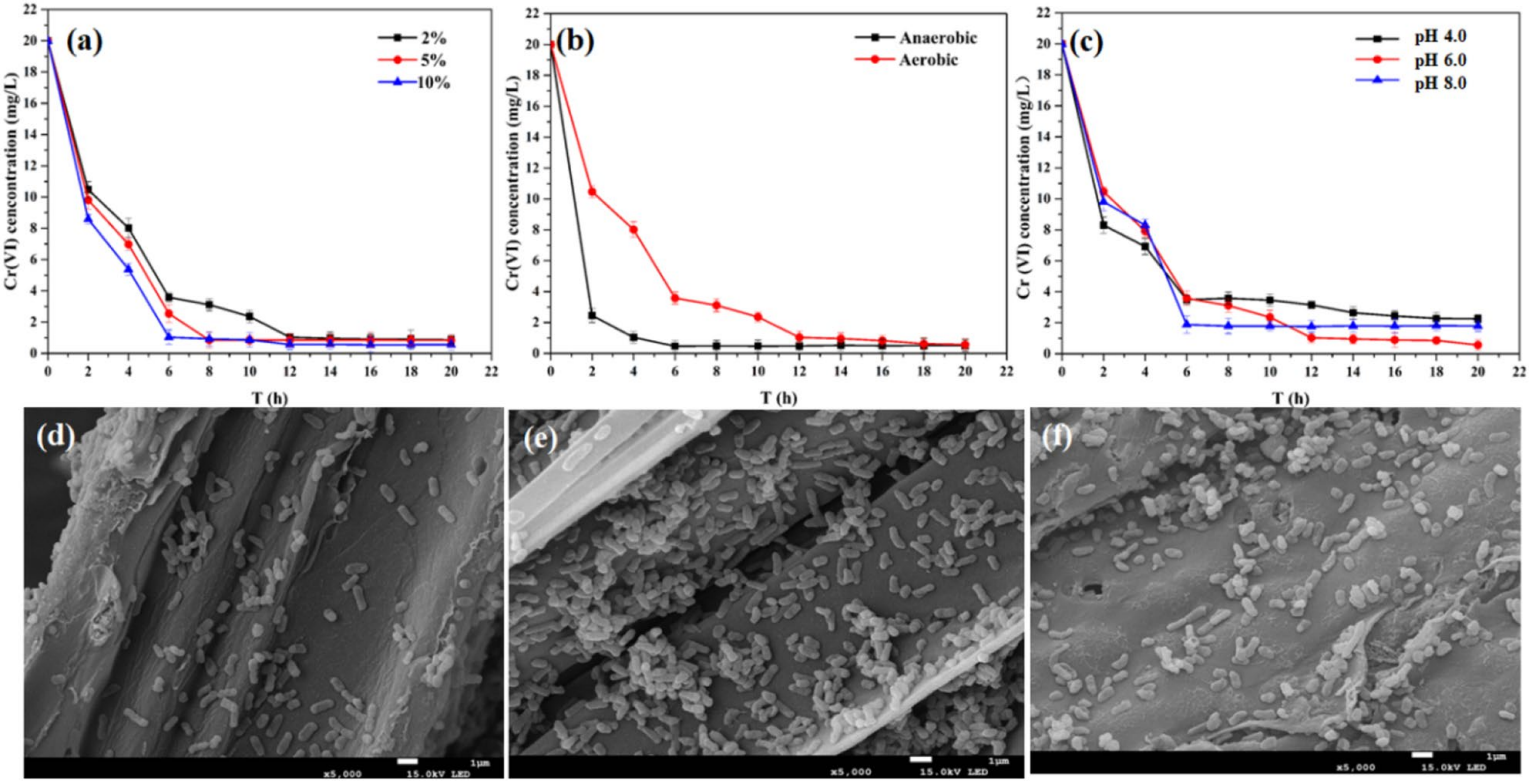
Effect of (**a**) inoculation amount, (**b**) oxygen condition, and (**c**) solution pH on Cr(VI) removal rate. Experimental conditions: Cr(VI) concentration 20 mg L^−1^, solution volume 100 mL and reaction time 24 h. (**d**–**f**) the stability of *K. variicola* H12-CMC-FeS@biochar under pH 4.0, pH 6.0, pH 8.0 respectively.

In this experiment, the *K. variicola* H12-CMC-FeS@biochar system was further studied. The effects of inoculation amount, oxygen, and pH on the *K. variicola* H12-CMC-FeS@biochar system to remove Cr (VI) are shown in Fig. 4. Figure 4a (Data provided in Table S8) showed that at the 6th hour of treatment, the inoculation amount of 10% (which had a removal rate of 95%) was higher than the inoculation amount of 2% (which had a removal rate of 81%). It indicated that the inoculation amount of *K. variicola* H12 had a certain effect on the efficiency of Cr(VI) removal by *K. variicola* H12-CMC-FeS@biochar system. The larger the inoculation amount, the higher the processing efficiency. These was consistent with the results of Liu et al. (2019) study^50^. Cr(VI) was removed more efficiently under anaerobic conditions, relative to aerobic conditions(Fig. 4b) (Data provided in Table S9). Under the condition of pH 6.0, the removal of Cr(VI) is slightly more efficient than pH 4.0, pH 8.0 (Fig. 4c) (Data provided in Table S10).These experiments showed that different environmental conditions (inoculation amount, oxygen, pH) can affect the efficiency of the *K. variicola* H12-CMC-FeS@biochar system in the treatment of Cr(VI), which needs to be considered in practical application. In addition, SEM analysis shown that there are some differences in the stability of *K. variicola* H12-CMC-FeS@biochar under different acidic and alkaline conditions (Fig. 4d–f). In the pH 4.0 and 8.0, the amount of *K. variicola* H12 was relatively small compared to that in the pH 6.0. Therefore, the *K. variicola* H12-CMC-FeS@biochar system performs well under the conditions of anaerobic, 10% inoculation amount and pH 6.0.

## Discussion

A groundwater remediation method for Cr (VI) removal using *K. variicola* H12-CMC-FeS@biochar was developed. CMC-FeS@biochar was successfully synthesized and utilized in the effective Cr (VI) removal from Cr(VI)-contaminated aqueous solution. *K. variicola* H12 can tolerate 100 mg L^−1^ of Cr(VI). The CMC-FeS@biochar increased the growth of *K. variicola* H12 at late logarithmic phase, though it decelerated the growth of *K. variicola* H12 at lag phase. With the addition of *K. variicola* H12, Cr(VI) removal rate of *K. variicola* H12-CMC-FeS@biochar system reached 96.6% for 10 h and the variety of surface functional groups increases, which provides more binding sites. *K. variicola* H12-CMC-FeS@biochar had a synergistic relationship with each other, but it was not a superposition of efficiency.

The optimal conditions for the growth of *K. variicola* H12 are pH 6.0 and 30 °C. Tryptone, yeast extract and NaCl used as nitrogen sources, carbon source and inorganic salt were used by *K. variicola* H12. When Cr (VI) concentration arrived at 200 mg L^−1^, the growth of *K. variicola* H12 strain was completely inhibited. XPS and FTIR analysis showed that CMC-FeS was successfully loaded on biochar. Then *K. variicola* H12-CMC-FeS@biochar was successfully constructed. In this system, the maximum OD value of *K. variicola* H12 was promoted by CMC-FeS@biochar from 1.39 to 1.57 and the removal rate of Cr(VI) was reached 96.6% at 10 h. This was shorter than the previous study (36 h is required to process chromium for microbial-biochar)^59^. FTIR analysis shown that *K. variicola* H12-CMC-FeS@biochar has more functional groups on the surface to provide bonding Site. Under different acidic and alkaline environments, EDS showed that the *K. variicola* H12-CMC-FeS@biochar system remained stable except for different strains (*K. variicola* H12 grew best under neutral conditions).

In this study, *K. variicola* was found to have the ability to remove chromium, which provided a theoretical basis for *K. variicola* H12 to be used in groundwater pollution of heavy metals. In addition to the existing traditional chemical treatment methods, combining microorganisms (*K. variicola* H12) with CMC-FeS@biochar can remove Cr(VI) more efficiently, which provides a new idea for groundwater treatment methods. At the same time, the biochar formed by pyrolysis of waste wheat straw was used as the main load material, which has advantages in environmental protection and economy. It has the potential for practical application.

## Supplementary Information

Supplementary Information
