# Supplementary material for: A high-efficiency Klebsiella variicola H12-CMC-FeS@biochar for chromium removal from aqueous solution
Source: Sci Rep. 2021 Mar 23;11:6611. doi: 10.1038/s41598-021-85975-z (PMC7988177; doi:10.1038/s41598-021-85975-z)
Supplement: Supplementary file 1 — Supplementary Information [file 41598_2021_85975_MOESM1_ESM.docx]

**Supporting Information**

**A high-effciency *Klebsiella variicola* H12-CMC-FeS@biochar for chromium removal from aqueous solution**

Runlan Yu^a,b^, Meilian Man^a^, Zhaojing Yu^a^，Xueling Wu^a,b^, Li Shen^a,b*^, Yuandong Liu^a,b^, Jiaokun Li^a,b^, Mingchen Xia^a^, Weimin Zeng^a,b*^

^a^School of Minerals Processing and Bioengineering Central South University, Changsha 410083 China

^b^Key Laboratory of Biometallurgy, Ministry of Education, Changsha 410083,China

**Pages: 16**

**Figures: 3**

**Tables: 10**

**Table S1(a-c)** **Optimization of energy substrate for *K. variicola* H12 growth**

**(a)** Carbon sources

| Carbon sources | C_6_H_12_O_6_, | C_12_H_22_O_11_, | (C_6_H_10_O_5_)_n_ | C_2_H_3_O_2_Na  •3H_2_O | yeast extrac |
| --- | --- | --- | --- | --- | --- |
| OD | 1.0205 | 0.935 | 0.8365 | 0.995 | 1.556 |

**(b)** Nitrogen sources

| Nitrogen sources | Beef Extract | Peptone | (NH4)_2_SO_4_, | CH_4_N_2_O | Tryptone |
| --- | --- | --- | --- | --- | --- |
| OD | 1.5515 | 1.5375 | 1.2995 | 0.733 | 1.556 |

| Organic salts | NaH_2_PO_4_ | KH_2_PO_4_ | NH_4_H_2_PO_4_ | Na_3_PO_4_ | Nacl |
| --- | --- | --- | --- | --- | --- |
| OD | 1.447 | 1.4795 | 1.483 | 1.3135 | 1.556 |

**(c)** Organic salts

**Table S2(a-b)** **Optimization pH and temperature of *K. variicola* H12 growth**

**(a)** pH

| pH | 2 | 4 | 6 | 8 | 10 |
| --- | --- | --- | --- | --- | --- |
| OD | 0.0705 | 0.7995 | 1.621 | 1.03 | 0.0455 |

**(b)** Temperature

| Temperature  （℃） | 20 | 30 | 40 | 50 | 60 |
| --- | --- | --- | --- | --- | --- |
| OD | 1.485 | 1.595 | 1.3203 | 1.097 | 0.0436 |

**Table S3** **Effect of Cr (Ⅵ) concentrations on the growth of *K. variicola* H12 in LB medium**

| **Cr(Ⅵ)**  **Time(h)** | **0**  **(mg L^-1^)** | **Error** | **20**  **(mg L^-1^)** | **Error** | **60**  **(mg L^-1^)** | **Error** | **100**  **(mg L^-1^)** | **Error** | **200**  **(mg L^-1^)** | **Error** |
| --- | --- | --- | --- | --- | --- | --- | --- | --- | --- | --- |
| 2 | 0.159 | 0.02 | 0.1425 | 0.045 | 0.1225 | 0.035 | 0.1124 | 0.048 | 0.1136 | 0.025 |
| 4 | 0.644 | 0.045 | 0.3215 | 0.038 | 0.1285 | 0.047 | 0.1145 | 0.029 | 0.1158 | 0.035 |
| 6 | 0.8245 | 0.035 | 0.419 | 0.06 | 0.1608 | 0.035 | 0.1168 | 0.035 | 0.1165 | 0.028 |
| 8 | 1.084 | 0.06 | 0.544 | 0.047 | 0.19023 | 0.048 | 0.11865 | 0.029 | 0.118 | 0.029 |
| 10 | 1.178 | 0.047 | 0.6985 | 0.035 | 0.19249 | 0.028 | 0.11793 | 0.034 | 0.1182 | 0.035 |
| 12 | 1.227 | 0.035 | 0.848 | 0.05 | 0.20986 | 0.034 | 0.12096 | 0.055 | 0.11806 | 0.031 |
| 14 | 1.287 | 0.04 | 0.9445 | 0.048 | 0.2635 | 0.04 | 0.12882 | 0.047 | 0.1181 | 0.0381 |
| 16 | 1.317 | 0.05 | 1.028 | 0.028 | 0.30848 | 0.028 | 0.15987 | 0.038 | 0.11807 | 0.028 |
| 18 | 1.3425 | 0.048 | 1.103 | 0.036 | 0.3512 | 0.055 | 0.17968 | 0.028 | 0.1179 | 0.026 |
| 20 | 1.3625 | 0.028 | 1.1595 | 0.045 | 0.45432 | 0.032 | 0.22764 | 0.052 | 0.11757 | 0.04 |
| 22 | 1.3705 | 0.05 | 1.1661 | 0.035 | 0.56505 | 0.04 | 0.25825 | 0.036 | 0.1178 | 0.032 |
| 24 | 1.37 | 0.035 | 1.185 | 0.033 | 0.65 | 0.048 | 0.267 | 0.035 | 0.118 | 0.034 |
| 26 | 1.3705 | 0.029 | 1.1852 | 0.048 | 0.651 | 0.033 | 0.2672 | 0.058 | 0.11598 | 0.029 |
| 28 | 1.3658 | 0.036 | 1.185 | 0.04 | 0.6525 | 0.029 | 0.2673 | 0.029 | 0.11453 | 0.031 |
| 30 | 1.3625 | 0.045 | 1.1753 | 0.029 | 0.65045 | 0.047 | 0.267 | 0.038 | 0.11381 | 0.041 |
| 32 | 1.32325 | 0.028 | 1.1705 | 0.033 | 0.60563 | 0.038 | 0.24069 | 0.042 | 0.10892 | 0.035 |
| 34 | 1.3209 | 0.035 | 1.16932 | 0.029 | 0.59421 | 0.06 | 0.24056 | 0.051 | 0.10858 | 0.028 |
| 36 | 1.32085 | 0.033 | 1.16856 | .0.055 | 0.5816 | 0.028 | 0.2405 | 0.045 | 0.1058 | 0.042 |

**Table S4 Cr (Ⅵ) reduction experiment by *K. variicola* H12**

| **Time(h)** | **Total chromium**  **( mg L^-1^)** | **Cr(Ⅵ)**  **( mg L^-1^)** | **Cr(Ⅲ)**  **( mg L^-1^)** |
| --- | --- | --- | --- |
| 0 | 20 | 20 | 0 |
| 2 | 17.64151 | 4.90566 | 12.73585 |
| 4 | 16.41509 | 8.20755 | 8.20754 |
| 6 | 13.11321 | 5.66038 | 7.45283 |
| 8 | 7.83019 | 2.64151 | 5.18868 |
| 10 | 8.39623 | 1.79245 | 6.60378 |
| 12 | 6.69811 | 1.59245 | 5.10566 |
| 14 | 3.77358 | 1.50943 | 2.26415 |

**Table S5** **Effect of CMC-FeS@biochar on the growth of *K. variicola* H12**

| **Time(h)** | ***K. variicola* H12** | **Error** | ***K. variicola* H12-CMC-FeS @ biochar** | **Error** |
| --- | --- | --- | --- | --- |
| 2 | 0.231 | 0.028 | 0.1595 | 0.023 |
| 4 | 0.3505 | 0.045 | 0.644 | 0.04 |
| 6 | 0.744 | 0.023 | 0.8245 | 0.045 |
| 8 | 1.0135 | 0.04 | 0.956 | 0.04 |
| 10 | 1.188 | 0.035 | 1.084 | 0.029 |
| 12 | 1.292 | 0.029 | 1.178 | 0.035 |
| 14 | 1.384 | 0.042 | 1.227 | 0.046 |
| 16 | 1.478 | 0.037 | 1.287 | 0.031 |
| 18 | 1.534 | 0.028 | 1.317 | 0.032 |
| 20 | 1.544 | 0.035 | 1.3425 | 0.025 |
| 22 | 1.543 | 0.0284 | 1.3305 | 0.029 |
| 24 | 1.545 | 0.042 | 1.345 | 0.035 |

**Table S6 Cr(Ⅵ) concentration on the growth of *K. variicola* H12**

| **Cr(Ⅵ)**  **(mg L^-1^)** | ***K. variicola* H12-CMC-FeS @ biochar** | **Error** | ***K. variicola* H12** | **Error** |
| --- | --- | --- | --- | --- |
| 20 | 48 | 0.8 | 24 | 1.2 |
| 60 | 12 | 1.5 | 8 | 0.9 |
| 100 | 8 | 1.36 | 7.2 | 1.23 |
| 200 | 6.8 | 1 | 4 | 1 |

**Table S7(a-b)** **Cr(VI) removal efficiency of H12, biochar, H12+biochar, FeS, H12+FeS, CMC-FeS@biochar, H12+CMC-FeS@biochar**

**(a)**

| **Time(h)** | **H12** | **Error** | **biochar** | **Error** | **H12+ biochar** | **Error** | **FeS** | **Error** |
| --- | --- | --- | --- | --- | --- | --- | --- | --- |
| 0 | 20 | - | 20 |  | 20 | - | 20 | - |
| 2 | 9.35849 | 0.392 | 16.69811 | 0.589 | 8.9434 | 0.392 | 12.20755 | 0.392 |
| 4 | 7.45283 | 0.498 | 12.54717 | 0.458 | 7.83019 | 0.498 | 11.32075 | 0.498 |
| 6 | 6.13208 | 0.485 | 11.79245 | 0.358 | 6.56604 | 0.485 | 9.15094 | 0.598 |
| 8 | 5.09434 | 0.412 | 9.62264 | 0.238 | 4.71698 | 0.212 | 9.4717 | 0.412 |
| 10 | 3.56604 | 0.325 | 8.96196 | 0.269 | 3.83019 | 0.325 | 7.54528 | 0.325 |
| 12 | 1.98563 | 0.45 | 8.35625 | 0.456 | 1.90566 | 0.45 | 5.36604 | 0.398 |
| 14 | 1.0569 | 0.364 | 7.65895 | 0.321 | 1.35849 | 0.264 | 5.09434 | 0.364 |
| 16 | 0.95689 | 0.348 | 7.18953 | 0.432 | 0.96196 | 0.248 | 4.95685 | 0.448 |
| 18 | 0.78569 | 0.4 | 6.98502 | 0.296 | 0.65283 | 0.4 | 4.52957 | 0.47 |
| 20 | 0.75472 | 0.385 | 6.98113 | 0.354 | 0.56604 | 0.285 | 4.5283 | 0.385 |

**(b)**

| **Time**  **(h)** | **H12+FeS** | **Error** | **CMC-FeS**  **@ biochar** | **Error** | **H12+CMC-FeS @ biochar** | **Error** |
| --- | --- | --- | --- | --- | --- | --- |
| 0 | 20 | - | 20 | - | 20 | - |
| 2 | 8.4717 | 0.295 | 10.18868 | 0.398 | 8.4717 | 0.392 |
| 4 | 6.22642 | 0.398 | 10.09434 | 0.358 | 5.03774 | 0.498 |
| 6 | 5.59434 | 0.486 | 9.90566 | 0.485 | 2.58491 | 0.585 |
| 8 | 4.92453 | 0.296 | 9.43396 | 0.412 | 1.61321 | 0.412 |
| 10 | 4.09434 | 0.425 | 7.65283 | 0.625 | 0.68966 | 0.325 |
| 12 | 2.55849 | 0.45 | 6.25895 | 0.55 | 0.61059 | 0.45 |
| 14 | 1.98868 | 0.314 | 4.98963 | 0.364 | 0.62366 | 0.364 |
| 16 | 0.84587 | 0.348 | 4.05286 | 0.448 | 0.5588 | 0.348 |
| 18 | 0.76359 | 0.412 | 3.58457 | 0.4 | 0.56965 | 0.4 |
| 20 | 0.75472 | 0.369 | 3.20755 | 0.375 | 0.56604 | 0.385 |

**Table S8** **Effect of inoculation amount on Cr(VI) removal rate**

| **Time(h)** | **2%** | **Error** | **5%** | **Error** | **10%** | **Error** |
| --- | --- | --- | --- | --- | --- | --- |
| 0 | 20 | - | 20 | - | 20 | - |
| 2 | 10.4717 | 0.525 | 9.81132 | 0.589 | 8.58491 | 0.296 |
| 4 | 8.01887 | 0.618 | 6.98113 | 0.398 | 5.37736 | 0.385 |
| 6 | 3.58491 | 0.2965 | 2.54717 | 0.564 | 1.03774 | 0.465 |
| 8 | 3.11321 | 0.3785 | 0.84906 | 0.483 | 0.92453 | 0.435 |
| 10 | 2.35693 | 0.3985 | 0.85066 | 0.296 | 0.86604 | 0.454 |
| 12 | 1.03688 | 0.164 | 0.85097 | 0.385 | 0.56804 | 0.324 |
| 14 | 0.95569 | 0.398 | 0.84866 | 0.4 | 0.57346 | 0.289 |
| 16 | 0.90865 | 0.325 | 0.86006 | 0.476 | 0.54669 | 0.468 |
| 18 | 0.89316 | 0.596 | 0.83956 | 0.321 | 0.54897 | 0.235 |
| 20 | 0.86604 | 0.248 | 0.83825 | 0.354 | 0.56379 | 0.36 |

**Table S9 Effect of oxygen condition on Cr(VI) removal rate**

| **Time(h)** | **Anaerobic**  **(mg L^-1^)** | **Error** | **Aerobic**  **(mg L^-1^)** | **Error** |
| --- | --- | --- | --- | --- |
| 0 | 20 | - | 20 | - |
| 2 | 2.45283 | 0.45 | 10.4717 | 0.392 |
| 4 | 1.03774 | 0.375 | 8.01887 | 0.498 |
| 6 | 0.4717 | 0.3582 | 3.58491 | 0.385 |
| 8 | 0.4857 | 0.352 | 3.11321 | 0.412 |
| 10 | 0.47693 | 0.385 | 2.35693 | 0.325 |
| 12 | 0.47896 | 0.298 | 1.03688 | 0.4 |
| 14 | 0.52069 | 0.365 | 0.95569 | 0.364 |
| 16 | 0.50266 | 0.412 | 0.8257 | 0.348 |
| 18 | 0.50569 | 0.456 | 0.6257 | 0.4 |
| 20 | 0.50604 | 0.3866 | 0.56604 | 0.385 |

**Table S10 Effect of solution pH on Cr(VI) removal rate**

| **Time(h)** | **pH 4** | **Error** | **pH 6** | **Error** | **pH 8** | **Error** |
| --- | --- | --- | --- | --- | --- | --- |
| 0 | 20 | - | 20 | - | 20 | - |
| 2 | 8.30189 | 0.525 | 10.4717 | 0.296 | 9.81132 | 0.589 |
| 4 | 6.92453 | 0.518 | 7.91887 | 0.385 | 8.30189 | 0.398 |
| 6 | 3.49057 | 0.2965 | 3.58491 | 0.465 | 1.88679 | 0.564 |
| 8 | 3.58491 | 0.3785 | 3.11321 | 0.435 | 1.79245 | 0.483 |
| 10 | 3.46529 | 0.3985 | 2.35693 | 0.454 | 1.78266 | 0.296 |
| 12 | 3.15699 | 0.164 | 1.03688 | 0.324 | 1.76359 | 0.385 |
| 14 | 2.6524 | 0.398 | 0.95569 | 0.289 | 1.80624 | 0.4 |
| 16 | 2.43866 | 0.325 | 0.89316 | 0.468 | 1.79259 | 0.476 |
| 18 | 2.28797 | 0.396 | 0.86604 | 0.235 | 1.80963 | 0.321 |
| 20 | 2.26415 | 0.248 | 0.56604 | 0.36 | 1.8057 | 0.354 |


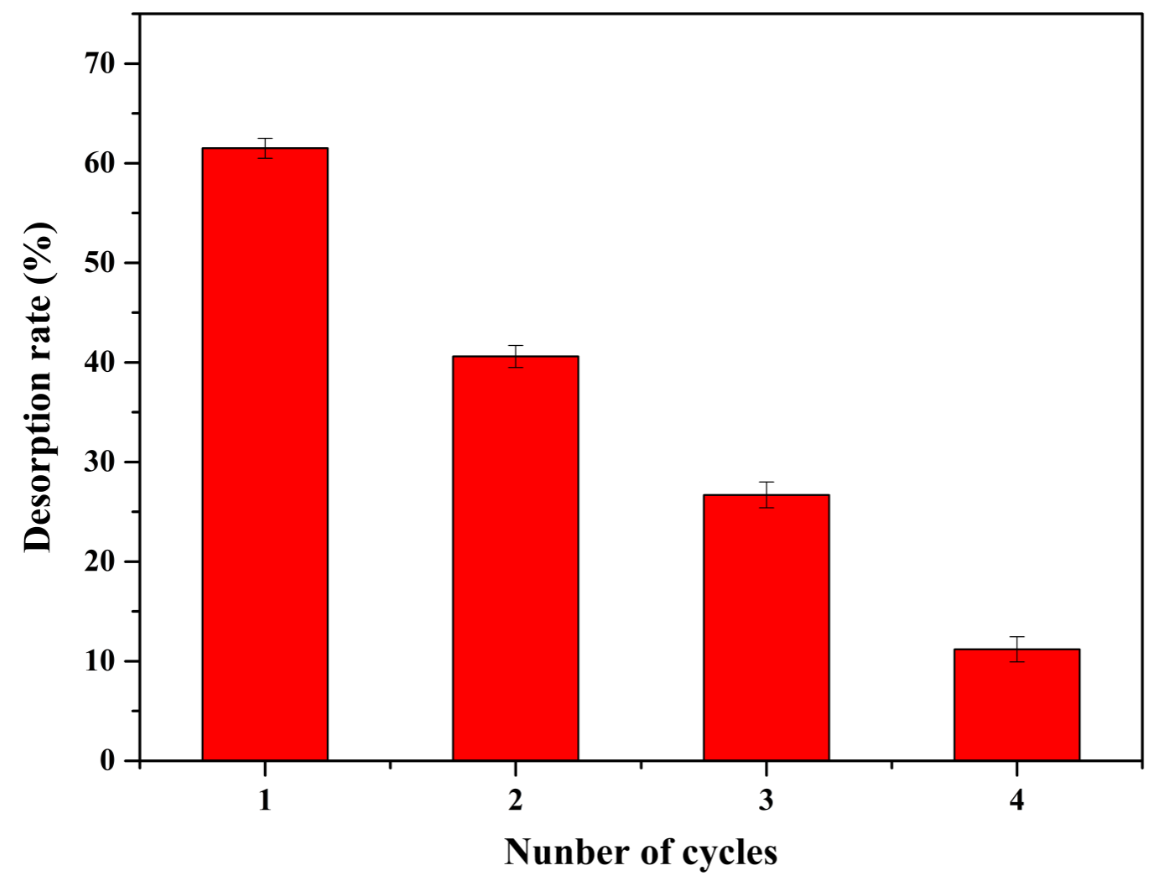


**Fig. S1** Regeneration study of *K. variicola* H12-CMC-FeS@biochar.


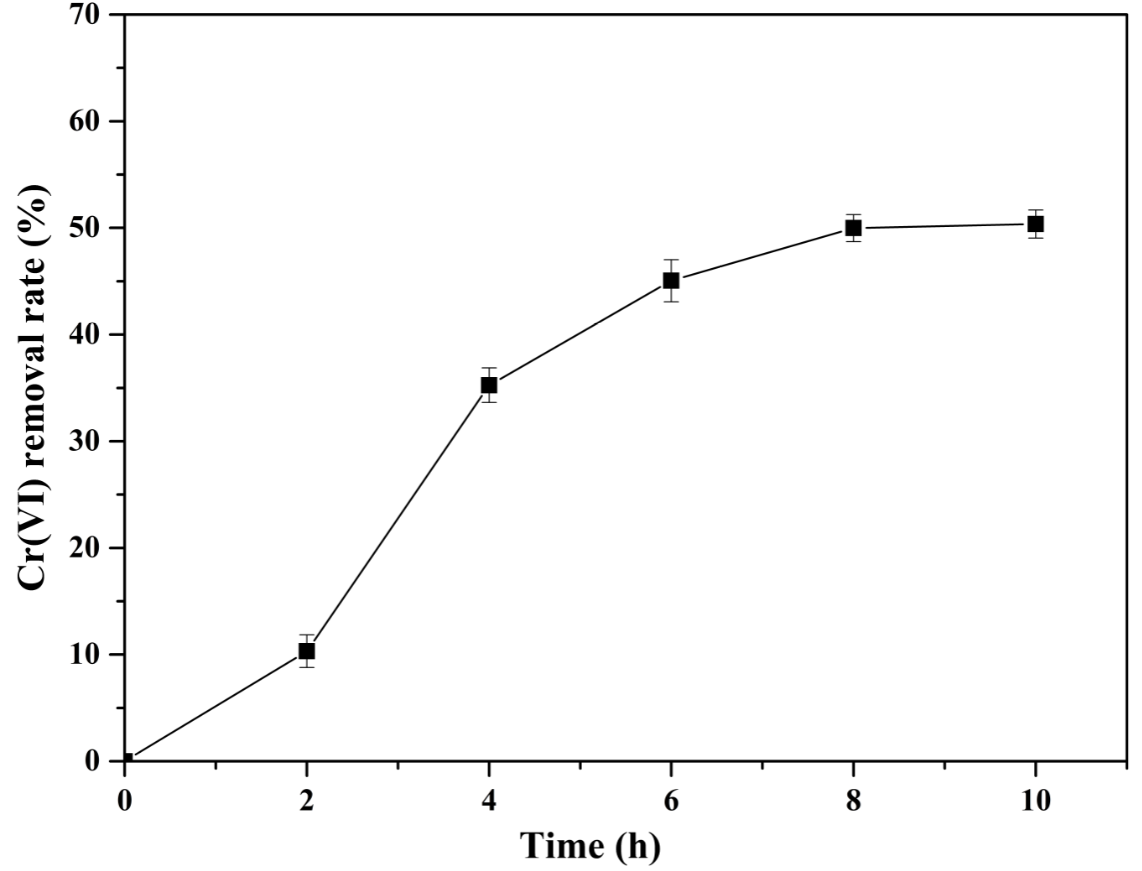


**Fig. S2** Removal rate of Cr(VI) by *K. variicola* H12-CMC-FeS@biochar in real water samples.


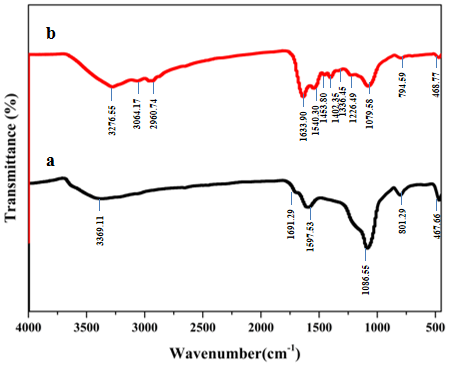


Fig. S3 FTIR spectra of biochar (a) Before and (b) after treatment of Cr(VI).
